# Effects of Aging Treatment on Corrosion Behavior of a Tensile Deformed Al-Cu-Mn-Fe-Zr Alloy in 3.5% NaCl Solution

**DOI:** 10.3390/ma14175062

**Published:** 2021-09-03

**Authors:** Qing Tian, Qiu-Mei Yang, Yong-Cheng Lin, Jun-Quan Wang, Xu-Hao Zhu

**Affiliations:** 1 Department of Electronic Science, Huizhou University, Huizhou 516007, China; jinhanlin830@sina.com; 2 School of Mechanical and Electrical Engineering, Central South University, Changsha 410083, China; Xianyangwu88@163.com (Q.-M.Y.); jinlongzhang1986@163.com (J.-Q.W.); mschen77@163.com (X.-H.Z.)

**Keywords:** alloy, aging, corrosion mechanism, microstructure

## Abstract

In this paper, the effects of an aging treatment on the corrosion resistance/mechanism of a tensile deformed Al-Cu-Mn-Fe-Zr alloy are investigated. The impedance magnitude and polarization resistance increase, while the corrosion current decreases with the increased aging time and temperature. The discontinuously-distributed precipitates and precipitation-free zone, which can cut the corrosion channels, appear at grain boundaries when the temperature is relatively high and the aging time is relatively long. They can improve the corrosion resistance. Additionally, the intergranular and pitting corrosion are the main mechanisms. The intergranular corrosion is likely to occur in an under-aged alloy. This is because the potential difference between the grain boundaries and grains is high, due to the segregation of Cu atoms. When the aging degree is increased, the grain boundary precipitates reduce the potential difference, and the intragranular precipitates make the surrounding matrix prone to dissolution. As such, the pitting corrosion is likely to occur in the over-aged alloys.

## 1. Introduction

Because of their outstanding mechanical and physical properties, aluminum alloys are widely used in the fields of ship building and aerospace [1,2,3,4]. Al-Cu alloys are often used in the production of high-performance components, such as rocket engines, and pressure vessels, etc. These components are thin-walled and large-sized, and usually formed by tensile deformation [5,6,7,8]. Al alloys have been also selected as the building material for space habitats and equipment. Generally, the service environment of the components is severe. The alloys or components should have excellent mechanical and corrosion properties [9,10,11,12,13]. Therefore, it is important to design suitable heat treatment methods to enhance the mechanical and corrosion properties of Al-Cu alloys.

In recent years, many scholars have studied the effects of aging on the precipitation kinetics [14,15], corrosion resistance [16,17,18], as well as some other mechanical properties of Al-Cu alloys [19,20,21,22,23,24]. However, the corrosion performance of Al-Cu alloys still needs comprehensive investigation. Lin et al. [25] and Li et al. [26] investigated the relationship between the corrosion behavior and microstructures of 2024Al alloy. In their study, de Sousa Araujo et al. [27] revealed the effects of thermomechanical treatments on the corrosion behavior of a 2198 Al–Cu–Li alloy. Wang et al. [28] discussed the effects of the S phase on the pitting initiation of an Al-Cu-Mg alloy. Ma et al. [29] significantly enhanced the corrosion resistance of 2219 Al alloy welds by laser surface melting. Liu et al. [30] indicated that the effect of microstructure refinement on corrosion performance is weak, but the effect of second phases is significant. Surekha et al. [31] found that the dissolution of the Al_2_Cu phase can improve the corrosion resistance of a 2219 Al alloy. Lu et al. [32] found that pre-deformation can increase the θ′ phase within grains and improve the corrosion resistance of a 2219 alloy. Huang et al. [33] found the phases at grain boundaries can reduce the intergranular corrosion sensitivity of an Al-Cu-Li alloy. Grilli et al. [34] indicated that the intermetallics act as a cathode in the corrosion process, dissolving the surrounding aluminum matrix. Chen et al. [35] found the multiaxial fatigue life decreases with the increased pre-corrosion time. Hikku et al. [36] improved the corrosion resistance by adding graphene nanocomposite coating and chromium plating on a 2219 Al alloy surface. Kairy et al. [37] found that adding Zr and Sc elements to Al-Cu alloys can reduce the corrosion current, thereby reducing the pitting/intergranular corrosion. Zhu et al. [38] improved the corrosion resistance of the alloy welds by heat treatment. Emarati et al. [39] improved the corrosion resistance of a 2024 Al alloy by electric etching, anodizing, sealing and surface chemical modification.

Although there is some research on the mechanical and corrosion properties of Al-Cu alloys, there are few studies on the effects of an aging treatment on the corrosion properties, as well as the corrosion mechanisms, of a deformed Al-Cu-Mn-Fe-Zr alloy. Furthermore, this topic is very important for industrial production components, such as thin-walled components produced by tensile deformation or spinning processing. In this study, a tensile deformed Al-Cu-Mn-Fe-Zr alloy is aged at 155-175 °C for 6–24 h. The electrochemical corrosion experiments were performed in a 3.5% NaCl solution. The electrochemical impedance spectroscopy (EIS) and polarization curves of the aged alloy are measured in order to discuss the effects of aging on corrosion resistance. To discuss corrosion mechanisms, the corrosion properties and corrosion morphology of the aged alloy are analyzed.

## 2. Materials and Experiments

### 2.1. Materials Preparation

**Figure 1:**
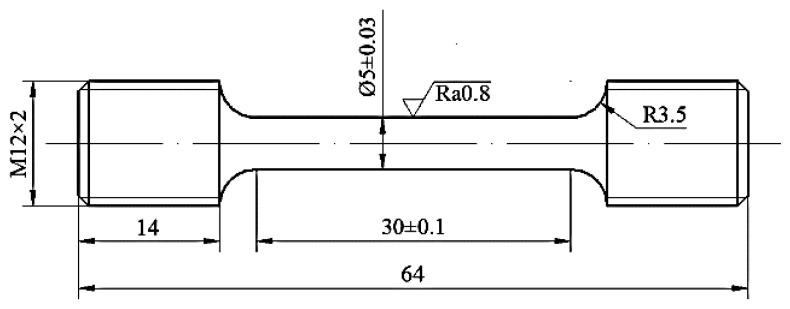
Specific dimensions of tensile test specimen (unit: mm).

A rolled Al-Cu-Mn-Fe-Zr alloy sheet with the chemical compositions of (Al-6.37Cu-0.30Mn-0.16Fe-0.12Zr-0.05Si-0.04Ti (wt.%)), was used. Firstly, the alloy sheet was solution-treated at the temperature and time of 535 °C and 40 min, respectively. The tensile test specimens were cut from the solution-treated sheet (in the rolling direction). According to the GB/T4338-2006 “Metal Material High Temperature Tensile Test Method”, the tensile test specimens meeting the requirements were then obtained through CNC machining, as shown in Figure 1. The specimens were then stretched on the MTS-GWT2105 testing machine at 150 °C, with a deformation amount of 15%, and tensile speed of 0.54 mm/min. Afterwards, the specimens were cooled to room temperature in the air. Finally, the deformed specimens were aged according to the designed conditions shown in Table 1.

### 2.2. Electrochemical Corrosion Experiments

The electrochemical corrosion experiments were performed on the CHI660E electrochemical workstation. The samples, with a length of 10 mm, were cut from the gauge segment of the aged alloy, and the impurities and oxide film were removed from surface by polishing. The surface area of the samples that were exposed to the 3.5% NaCl solution was 40 mm^2^. Firstly, the samples were immersed in solution for 10 min to obtain open circuit potential. The electrochemical impedance spectroscopy (EIS) measurements were then conducted with an alternating current (AC) signal, of which the frequency ranged from 0.01 Hz to 100 KHz and the amplitude was 10 mV. In addition, the preparation of the samples for the polarization curve tests are consistent with the EIS test. The scan interval was selected according to the open circuit potential. Based on previous research [29], the open circuit potential of a 2219 Al alloy is between −0.6 V/SCE and −0.8 V/SCE. The scanning interval was −1.2~0 V/SCE, and scanning rate was 0.5 mV s^−1^.

### 2.3. Corrosion Morphology Observation

In order to investigate the effects of the aging treatment on corrosion morphology, a scanning electron microscope (SEM) (FEI Electron Optics B.V; Prague, Czech Republic), optical microscope (OM), and laser scanning confocal microscope (LSCM) were used. Before the SEM and LSCM tests, the non-working surface of specimens was polished to ensure flatness, and the samples were vacuum sealed to avoid the oxidation of corrosion products. In order to analyze the corrosion depth of the longitudinal section of the corroded sample, the OM tests were conducted on an optical microscope (Olympus DSX500) (Olympus Corporation, Tokyo, Japan). Before the OM tests, the longitudinal profile was mechanically polished until the surface had no obvious scratches. In addition, a transmission electron microscope (TEM) (Tecnai G2 F20; FEI Company; Hillsboro, OR, USA) was used to observe precipitated phases in the aged alloy. Here, a TEM sample was obtained by mechanical grinding and stamping. Its thickness was 50–80 μm and the diameter was 3 mm. Afterwards, the samples were electropolished by double-jet in HNO_3_ (30%) and CH_3_OH (70%) at −25 °C.

## 3. Results and Discussion

### 3.1. Effect of Aging Parameters on Electrochemical Impedance Spectroscopy

**Figure 2:**
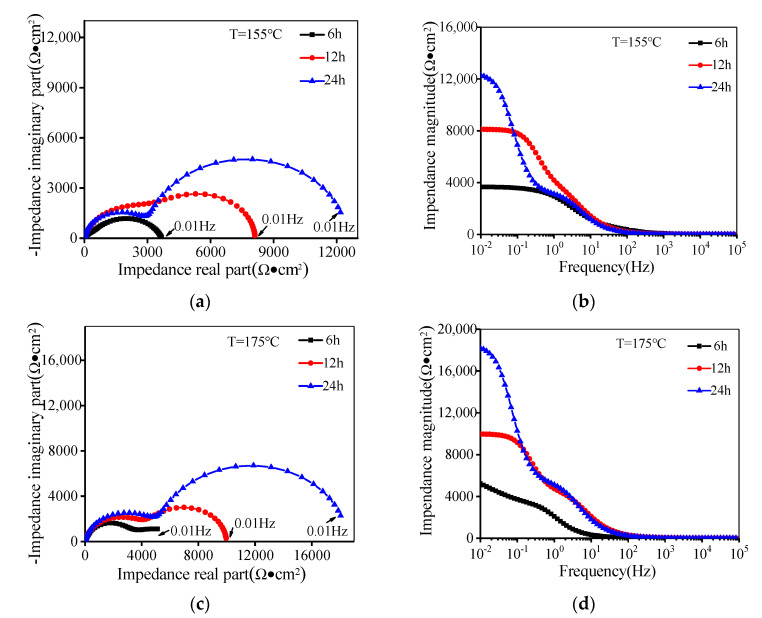
Electrochemical impedance spectroscopy: (**a**) Nyquist plots (T = 155 °C); (**b**) Bode diagrams (T = 155 °C);.(**c**) Nyquist plots (T = 175 °C); (**d**) Bode diagrams (T = 175 °C).

Figure 2 shows the EIS of the aged alloy. In Figure 2a, the curve has an obvious impedance arc at 155 °C for 6 h. When the aging time is 12 h or 24 h, two impedance arcs appear at the frequency of 0.01–1 Hz or 1–1000 Hz, respectively. The arc at the low frequency is mainly caused by the electrochemical corrosion process, while the arc at the high frequency is related to the charge transfer resistance at the interfaces between the electrolyte solution and alloy [40,41]. Generally, a large impedance arc indicates a high impedance magnitude. The impedance magnitude reflects the charge transfer ability of the sample surface. The higher impedance magnitude indicates that the electrode reaction is slower and the corrosion resistance is larger [42,43,44]. As shown in Figure 2b, the impedance magnitude increases with the extended aging time at a low frequency, showing that the corrosion resistance of the alloy is improved. As shown in Figure 2c,d, the effect of the aging time on the impedance magnitude is consistent with those shown in Figure 2a,b, which further indicates that the corrosion resistance becomes larger with the increased aging time. In Figure 2a,c, the radius of the impedance arc increases as the temperature is raised from 155 °C to 175 °C for the same aging time. Meanwhile, in Figure 2b,d, the impedance magnitude also shows the same rule, which indicates that the corrosion resistance increases with the raised aging temperature.

### 3.2. Establishment of Equivalent Circuit

**Figure 3:**
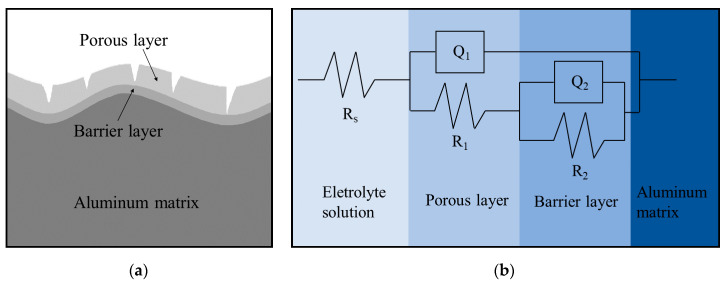
Schematic diagram of: (**a**) surface structure; (**b**) equivalent circuit.

The interface between the alloy and electrolyte solution is generally composed of oxide film, which includes two layers. The outer layer has many small holes, while the inner layer is dense, as shown in Figure 3a [44]. According to the characteristics of oxide film, it can be divided into a porous layer and a barrier layer [45,46] (Figure 3a). The alloy and electrolyte solution can be simplified into an equivalent circuit. In this work, the R(Q(R(QR))) model (Figure 3b) is used. R_s_ represents the resistance of the electrolyte solution, and its value mainly depends on the concentration of the electrolyte solution and the distance between the electrodes. R_1_ is the resistance of the porous layers. Additionally, R_2_ represents the charge transfer resistance, which corresponds to the barrier layers of oxide film. The constant phase element (Q) is used to describe the nonideal capacitance due to non-uniformed oxide film. Q_1_ and Q_2_ are the constant phase elements of the porous and the barrier layers, respectively. The impedance of constant phase elements can be expressed as [47],

(1) $$Z=Y^{-1}(j\omega {)}^{-n}$$

where *Y* represents the capacitance, *ω* is the angular frequency, n is the dispersion value of the constant phase element, and its value is between −1 and 1. When −1 < n < 0, 0 < n < 0.5, and 0.5 < n <1, the constant phase element is close to the inductance, resistance, and capacitance, respectively.

The electrochemical parameters are obtained by ZsimpWin software (v3.60), as shown in Table 2. R_s_ varies little at different conditions, and its value range is 5–11 $\left(\Omega \cdot {\mathrm{cm}}^{2}\right)$. As mentioned above, the value of R_s_ is mainly related to the concentration of the electrolyte solution and the distance between the electrodes. Therefore, the aging process has little effect on R_s_. Meanwhile, there is no obvious law in Q_1_ and Q_2_ at different conditions. The phenomenon shows that the oxide film is relatively non-uniform The dispersion values of the constant phase elements (n) are all above 0.5, and some values are 1, indicating its electrochemical characteristics are close to the ideal capacitance. However, n_2_ is 0.596 for the alloy aged at 155 °C for 6 h, which means that the charge diffusion occurs during the electrochemical corrosion.

**Figure 4:**
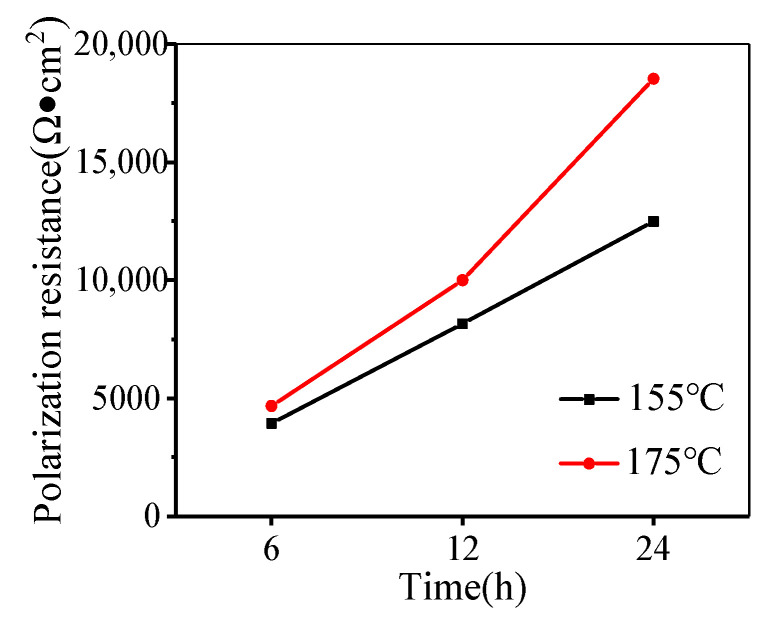
Polarization resistance (R_p_) under different aging conditions.

The sum of R_1_ and R_2_ is the polarization resistance (R_p_), which can represent the corrosion resistance of the studied alloy. Generally, it is believed that the corrosion current during electrochemical corrosion decreases with the increased R_p_, i.e., the corrosion resistance becomes increasingly larger. The values of R_p_ are shown in Figure 4. It can be observed the polarization resistance increases with the increased aging time and temperature.

### 3.3. Effect of Aging Parameters on Polarization Curves

**Figure 5:**
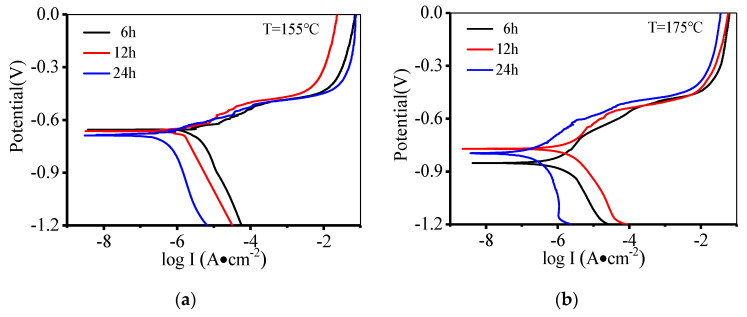
Polarization curves at: (**a**) T = 155 °C; (**b**) T = 175 °C.

Figure 5 shows the polarization curves of the aged alloy. The polarization curves are similar under different aging conditions, and there is no obvious passivation. This is because Cl^-^ is adsorbed on the surface of the alloy, and the formation of the passivation film is hindered [48,49,50]. The values of corrosion potential, corrosion current, as well as the slope of cathode and anode, are measured using the Tafel extrapolation method [45,51], as shown in Table 3. The corrosion potential decreases slightly with the increased aging time at 155 °C. When the aging temperature is raised to 175 °C, the corrosion potential changes irregularly at a different aging time. The corrosion potential reflects the possibility of electrochemical corrosion of the alloy [52,53,54], but the law of corrosion potential change is not obvious under the tested conditions. Generally, the corrosion dynamic parameters are used to characterize the corrosion resistance of alloys. Moreover, the corrosion current belongs to the category of corrosion dynamic parameters [55]. As such, this research selects the corrosion current as the judgment standard. The corrosion current is reduced by 90.23% at 175 °C when the aging time is increased from 6 h to 24 h. The corrosion current is reduced from 0.394 to 0.183 (i.e., a decrease of 53%), as the aging temperature is raised from 155 to 175 °C at the aging time of 24 h. In summary, the corrosion current decreases with the raised temperature and aging time, i.e., the corrosion resistance of the alloy increases.

### 3.4. Corrosion Morphology Analysis

**Figure 6:**
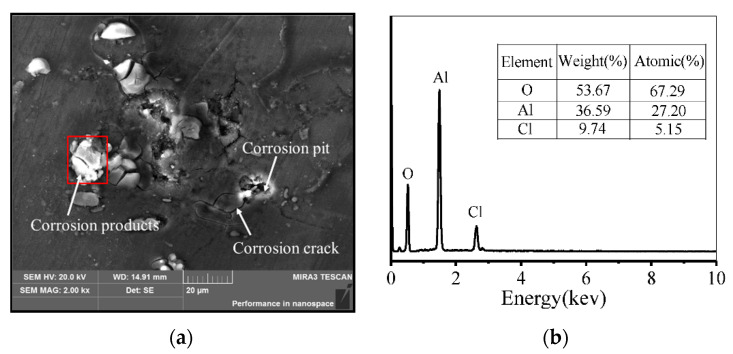
Corrosion morphology and corrosion products: (**a**) SEM observation; (**b**) EDS of corrosion products.

Figure 6 shows the corrosion morphology and corrosion products after electrochemical tests. In Figure 6a, many corrosion pits are distributed on the surface of the alloy, and the depth of corrosion is relatively larger. Meanwhile, some off-white corrosion products and corrosion cracks appear around the corrosion pits. In Figure 6b, the main elements of the corrosion products are measured as oxygen, aluminum, and chlorine, i.e., Al(OH)_3_ and AlCl_3_. The formation of Al(OH)_3_ is mainly due to the hydrolysis reaction, which is caused by some aluminum ions [56]. The hydrolysis reaction can be expressed as,

(2) $${\mathrm{Al}}^{3+}+3H_{2}O\to \mathrm{Al}{\left(\mathrm{OH}\right)}_{3}+3H^{+}$$

The formation of AlCl_3_ can be expressed as,

(3) $$\mathrm{Al}{\left(\mathrm{OH}\right)}_{3}{+\mathrm{Cl}}^{-}\to \mathrm{Al}{\left(\mathrm{OH}\right)}_{2}{\mathrm{Cl}+\mathrm{OH}}^{-}$$

(4) $$\mathrm{Al}{\left(\mathrm{OH}\right)}_{2}{\mathrm{Cl}+\mathrm{Cl}}^{-}\to \mathrm{Al}\left(\mathrm{OH}\right){\mathrm{Cl}}_{2}{+\mathrm{OH}}^{-}$$

(5) $$\mathrm{Al}\left(\mathrm{OH}\right){\mathrm{Cl}}_{2}{+\mathrm{Cl}}^{-}\to {\mathrm{AlCl}}_{3}{+\mathrm{OH}}^{-}$$

Corrosion products accumulate on the surface of the alloy during the electrochemical reaction, which inhibits the diffusion of dissolved Al^3+^. Non-diffusible Al^3+^ accumulates on the corrosion products, forming a strong electric field [57,58]. Thus, Cl^−^ accumulates to the outer layer of the corrosion products. Cl^−^ can continue to move into the corrosion products due to strong permeability. The corrosion products gradually crack under the combined influence of Cl^−^ and H^+^, and the matrix under the corrosion product continues to be corroded. The corrosion then mainly proceeds along the crack of the corrosion products.

**Figure 7:**
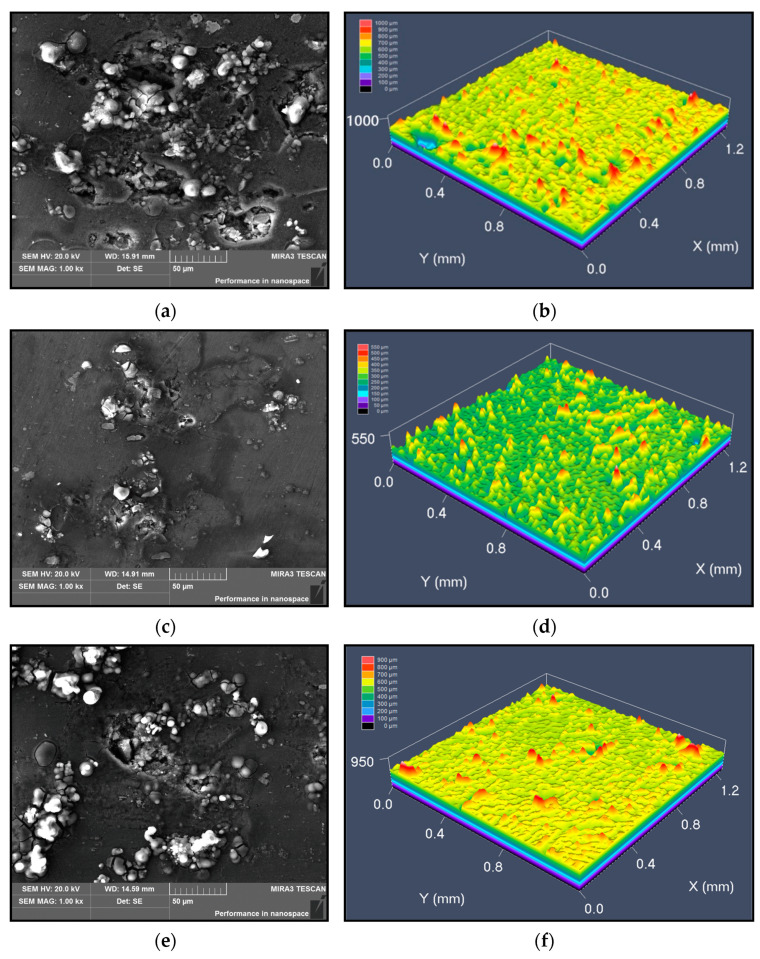

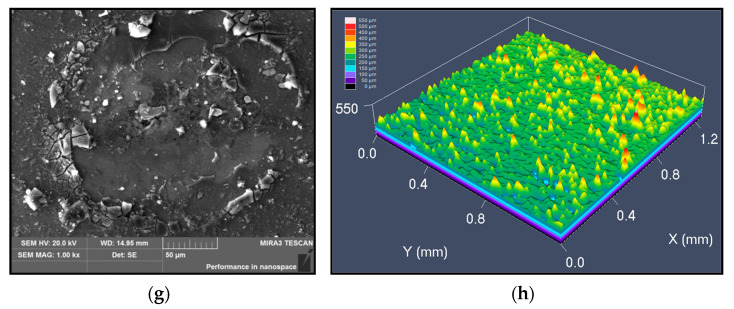
Corrosion morphology and laser confocal maps at: (**a**,**b**) T = 155 °C, *t* = 6 h; (**c**,**d**) T = 155 °C, *t* = 24 h; (**e**,**f**) T = 175 °C, *t* = 6 h; (**g**,**h**) T = 175 °C, *t* = 24 h.

**Figure 8:**
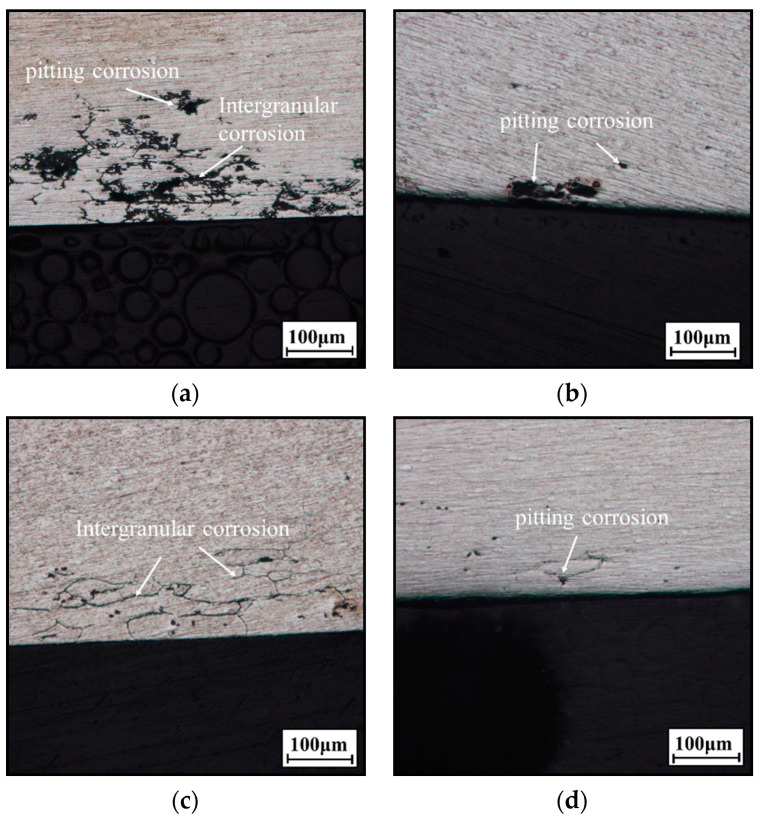
OM images of longitudinal section of the alloys at: (**a**) T = 155 °C, *t* = 6 h; (**b**) T = 155 °C, *t* = 24 h; (**c**) 175 °C, *t* = 6 h; (**d**) 175 °C, *t* = 24 h.

Figure 7 shows the corrosion morphology and laser confocal maps of the alloy. In Figure 7a,b, a large amount of corrosion products are accumulated on the surface, and the maximum height of the corrosion products reaches 1000 μm. As the aging time is extended to 24 h, the maximum height of the corrosion products decreases to 550 μm, as depicted in Figure 7c,d. In Figure 7e,f, many off-white corrosion products are distributed around the corrosion pits for the alloy aged at 175 °C for 6 h, and the corrosion cracks mainly propagate along the phase boundary. The laser confocal map shows that the maximum height of corrosion products is 900 μm. In Figure 8g,h, corrosion pits are only found in the center of the surface, and the maximum height of corrosion products decreases to 550 μm.

OM images of the longitudinal section of the alloy are shown in Figure 8. Generally, the main corrosion types of Al-Cu alloys are intergranular and pitting corrosion, and the corrosion type is affected by the microstructures [59,60,61,62,63]. In Figure 8a, the main type of corrosion is intergranular corrosion for the alloy aged at 155 °C for 6 h. The corrosion area and the corrosion depth are large-, and the maximum corrosion depth is 196 μm. In addition, the pitting corrosion can be observed at grain boundaries and within grains. As the aging time is increased to 24 h (Figure 8b), only the pitting corrosion is observed, and the maximum corrosion depth is 32 μm. In Figure 8a,c, the corrosion area and depth significantly decrease as the temperature is raised from 155 to 175 °C. As presented in Figure 8d, a few pits appear on the surface of the alloy aged at 175 °C for 24 h. The above results indicate that the corrosion type changes from the intergranular corrosion to the pitting corrosion with increasing the aging time and temperature. Meanwhile, the corrosion resistance is also improved with the raised temperature and aging time.

### 3.5. Corrosion Mechanism Analysis

**Figure 9:**
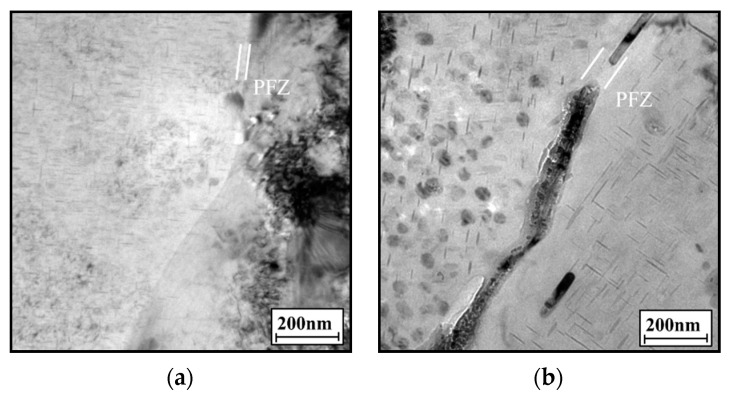

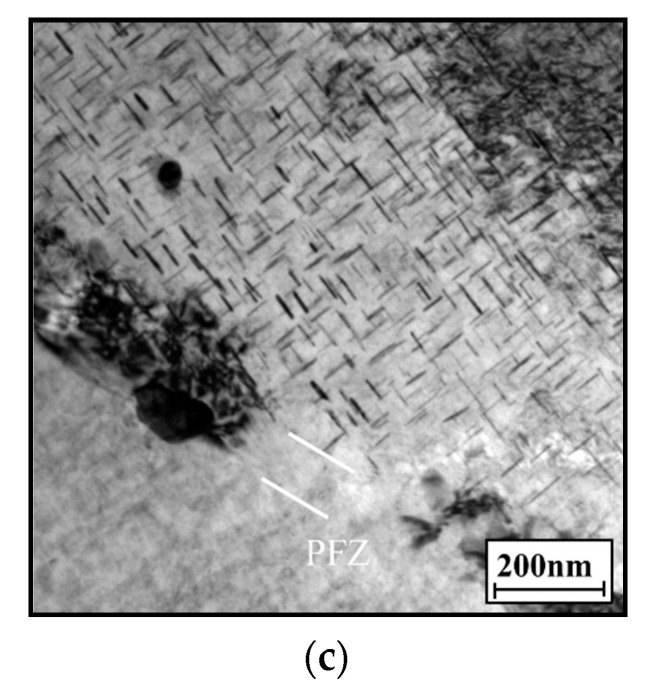
TEM images of grain boundaries at: (**a**) T = 155 °C, *t* = 6 h; (**b**) T = 155 °C, *t* = 24 h; (**c**) T = 175 °C, *t* = 24 h.

**Figure 10:**
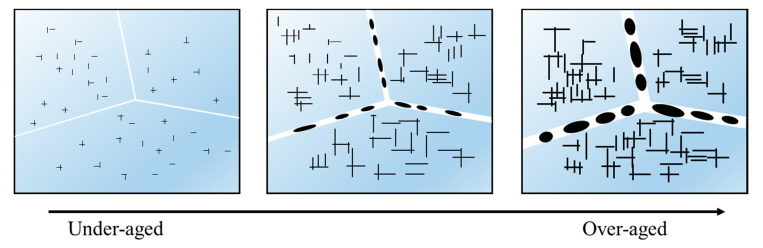
Schematic diagram of microstructure evolution.

Based on the above experimental results, the main corrosion types of the aged alloy are intergranular and pitting corrosion [64]. Intergranular corrosion is likely to occur in under-aged alloys, while the pitting corrosion likely appears in over-aged alloys. Corrosion types are mainly related to the continuity of precipitation phases and the precipitate-free zone (PFZ) at the grain boundaries [65,66,67,68]. Figure 9 shows TEM images of grain boundaries of the aged alloy. In Figure 9a, there is no precipitate at the grain boundaries and the PFZ is narrow for the alloy aged at 155 °C for 6 h. The grain boundary can accelerate the intergranular corrosion in the corrosion process. This is because the potential difference between the grain boundaries and grains is high, due to the segregation of Cu atoms. As the aging time is increased to 24 h (Figure 9b), the discontinuously-distributed precipitates appear at grain boundaries, and there are fine precipitates in the grains. The nucleation and growth of grain boundary precipitates consume a lot of Cu atoms, which reduces the saturation of solute atoms and promotes the increase of PFZ width. In Figure 9c, the grain boundary precipitates and intragranular precipitates become coarser, and the PFZ becomes wider in the alloy aged at 175 °C for 24 h. Figure 10 shows the schematic diagram of the evolution of the microstructures. When the alloy is under-aged, the intragranular precipitates are relatively fine, and there are flat PFZs at the grain boundaries. The potential difference between the grain boundaries and grains is high. A coherent circuit is then formed. As such, the alloy exhibits the high sensitivity to intergranular corrosion. When the alloy is over-aged, PFZ becomes more and more wide. In addition, the grain boundary precipitates and intragranular precipitates become more and more coarse. The potential difference decreases and intragranular precipitates make the surrounding matrix prone to dissolution. Moreover, the grain boundary precipitates and PFZ cut corrosion channels in the corrosion process, reducing the corrosion sensitivity of grain boundaries [45,54,69,70]. Thus, the pitting corrosion is more likely to occur in over-aged alloys. Additionally, the wider PFZ can protect the matrix from corrosion and improve the corrosion resistance of the alloy.

## 4. Conclusions

The effects of aging treatment on the corrosion resistance/mechanism of a tensile deformed Al-Cu-Mn-Fe-Zr alloy are investigated. Several important conclusions are shown below:

1. The impedance magnitude and polarization resistance increase, while the corrosion current decreases with increasing the aging time and temperature. Therefore, the corrosion resistance is improved.
2. The discontinuously-distributed precipitates and precipitation-free zone, which can cut the corrosion channels, appear at grain boundaries when the temperature is relatively high, and the aging time is relatively long. The corrosion sensitivity of grain boundaries can then be reduced.
3. The intergranular corrosion is likely to occur in under-aged alloys. This is because that the potential difference between the grain boundaries and grains is high due to the segregation of Cu atoms. When the aging degree is increased, the grain boundary precipitates reduce the potential difference, and the intragranular precipitates make the surrounding matrix prone to dissolution. As such, the pitting corrosion is more likely to occur in over-aged alloys.

## Figures and Tables

**Table 1:** Aging treatment parameter.

Case ID	Temperature (°C)	Time (h)
1	155	6
2		12
3		24
4	175	6
5		12
6		24

**Table 2:** Parameter fitting results of equivalent circuits.

Temperature (°C)	Time (h)	R_S_	Q_1_	n_1_	R_1_	Q_2_	n_2_	R_2_
		$\left(\Omega \cdot {\mathrm{cm}}^{2}\right)$	$\left(\mu F\cdot {\mathrm{cm}}^{-2}\right)$		$\left(\Omega \cdot {\mathrm{cm}}^{2}\right)$	$\left(\mu F\cdot {\mathrm{cm}}^{-2}\right)$		$\left(\Omega \cdot {\mathrm{cm}}^{2}\right)$
	6	6.435	0.89	1	0.123	48.42	0.596	3932
155	12	5.92	16.43	0.874	4523	10.84	1	3638
	24	5.14	15.91	0.8	34.13	276.9	0.8	9077
175	6	10.4	73.59	0.761	59.6	23.3	0.933	4617
	12	7.14	9.503	0.904	4930	1397	0.994	5072
	24	8.633	11.62	0.909	5785	203.9	0.994	12,750

**Table 3:** Corrosion parameters of the alloy under different aging conditions.

Temperature	Time	Corrosion Potential	Corrosion Current
(°C)	(h)	(V)	$\left({\mu A/\mathrm{cm}}^{2}\right)$
155	6	−0.655	2.747
	12	−0.663	1.757
	24	−0.688	0.394
175	6	−0.852	1.874
	12	−0.771	1.617
	24	−0.796	0.183

## Data Availability

The raw/processed data required to reproduce these findings cannot be shared at this time as the data also forms part of an ongoing study.

## References

[1] Tomczyk A., Seweryn A. (2017). Fatigue life of EN AW-2024 alloy accounting for creep pre-deformation at elevated temperature. Int. J. Fatigue.

[2] Lin Y.-C., Luo S.-C., Yin L.-X., Huang J. (2018). Microstructural evolution and high temperature flow behaviors of a homogenized Sr-modified Al-Si-Mg alloy. J. Alloy. Compd..

[3] Chen Y., Liu C., Zhou J., Wang F. (2019). Effect of alternate corrosion factors on multiaxial low-cycle fatigue life of 2024-T4 aluminum alloy. J. Alloy. Compd..

[4] Li Y., Shi Z., Lin J., Yang Y.-L., Rong Q. (2017). Extended application of a unified creep-ageing constitutive model to multistep heat treatment of aluminium alloys. Mater. Des..

[5] Lin Y.-C., Wu Q., He D.-G., Zhu X.-H., Liu D.-Y., Li X.-H. (2020). Effects of solution time and cooling rate on microstructures and mechanical properties of 2219 Al alloy for a larger spun thin-wall ellipsoidal head. J. Mater. Res. Technol..

[6] Murty S.V.S.N., Manwatkar S.K., Narayanan P.R. (2015). Role of metallographic analysis in the identification of location of crack initiation in a burst tested AA 2219 propellant tank. Metallogr. Microstruct. Anal..

[7] Li G.-A., Ma Z., Jiang J.-T., Shao W.-Z., Liu W., Zhen L. (2020). Effect of pre-stretch on the precipitation behavior and the mechanical properties of 2219 Al alloy. Materials.

[8] Zhu X.-H., Lin Y.-C., Wu Q., Jiang Y.-Q. (2020). Effects of aging on precipitation behavior and mechanical properties of a tensile deformed Al-Cu alloy. J. Alloy. Compd..

[9] Caltagirone P.E., Wheeler R.W., Benafan O., Bigelow G., Karaman I., Calkins F.T., Kuntz M.L., Leal P.B.C., Nicholson D.E., Ozcan H. (2021). Shape memory alloy-enabled expandable space habitat—Case studies for second CASMART student design challenge. Shape Mem. Superelast..

[10] Suita Y., Takai D., Sugiyama S., Terajima N., Tsukuda Y., Fujisawa S., Imagawa K. (2005). Welding experiments of aluminum alloy by space GHTA welding at ISS orbital pressure. Trans. Jpn. Soc. Aeronaut. Space Sci..

[11] Agafonov R., Vilkov F., Kasitsyn A., Predko P., Marchenkov A. (2016). Aluminum based alloys with rare-earth metals additives application for rocket-and-space engineering. Vestn. Mosk. Aviat. Inst..

[12] Lin Y.-C., Wang Z.-W., He D.-G., Zhou Y., Chen M.-S., Huang M.-H., Zhang J.-L. (2016). Effects of pre-treatments on precipitate microstructures and creep-rupture behavior of an Al-Zn-Mg-Cu alloy. J. Mater. Res..

[13] Lin Y.-C., Liu G., Chen M.-S., Zhang J.-L., Chen Z.-G., Jiang Y.-Q., Li J. (2016). Corrosion resistance of a two-stage stress-aged Al-Cu-Mg alloy: Effects of external stress. J. Alloys Compd..

[14] Chen M.-C., Wen M.-C., Chiu Y.-C., Pan T.-A., Tzeng Y.-C., Lee S.-L. (2020). Effect of natural aging on the stress corrosion cracking behavior of A201-T7 aluminum alloy. Materials.

[15] Xu J., Deng Y.L., Chen J. (2020). Enhancing the corrosion resistance of Al-Cu-Li alloys through regulating precipitation. Materials.

[16] Härtel M., Frint P., Abstoss K.G., Wagner M.F.-X. (2018). Effect of creep and aging on the precipitation kinetics of an Al-Cu alloy after one pass of ECAP. Adv. Eng. Mater..

[17] Lin Y.-C., Zhang J.-L., Liu G., Liang Y.-J. (2015). Effects of pre-treatments on aging precipitates and corrosion resistance of a creep-aged Al-Zn-Mg-Cu alloy. Mater. Des..

[18] Lin Y.-C., Xia Y.-C., Jiang Y.-Q., Zhou H.-M., Li L.-T. (2013). Precipitation hardening of 2024-T3 aluminum alloy during creep aging. Mater. Sci. Eng. A.

[19] Lin Y.-C., Liu G., Chen M.-S., Li J., Zhou M., Zhou H.-M. (2015). Effects of two-stage creep-aging processing on mechanical properties of an Al-Cu-Mg alloy. Mater. Des..

[20] Prabhu T.R. (2017). Correlation of deformation strain with the tensile properties and fracture mode of the AA 2014 alloy using simulations and experiments. J. Test. Eval..

[21] Santecchia E., Cabibbo M., Ghat M., Regev M., Spigarelli S. (2020). Physical modeling of the creep response of an Al-Cu-Mg alloy with a fine microstructure transformed by friction stir processing. Mater. Sci. Eng. A.

[22] Li G., Zhou L., Luo L., Wu X., Guo N. (2019). Microstructural evolution and mechanical properties of refill friction stir spot welded alclad 2A12-T4 aluminum alloy. J. Mater. Res. Technol..

[23] Wang W.G., Wang G., Guo G.N., Rong Y.M. (2018). Competitive relationship between thermal effect and grain boundary precipitates on the ductility of an as-quenched Al-Cu-Mn alloy. Int. J. Damage Mech..

[24] Fernández R., Bokuchava G., Toda-Caraballo I., Bruno G., Turchenko V., Gorshkova J., González-Doncel G. (2020). Analysis of the combined strengthening effect of solute atoms and precipitates on creep of aluminum alloys. Adv. Eng. Mater..

[25] Lin Y.-C., Jiang Y.-Q., Xia Y.-C., Zhang X.-C., Zhou H.-M., Deng J. (2014). Effects of creep-aging processing on the corrosion resistance and mechanical properties of a typical Al-Cu-Mg alloy. Mater. Sci. Eng. A.

[26] Li Y.-D., Zhang Y., Li S.-M., Zhao P.-Z. (2016). Influence of adipic acid on anodic film formation and corrosion resistance of 2024 aluminum alloy. Trans. Nonferr. Met. Soc..

[27] Araujo J.V.d., Milagre M.X., Ferreira R.O., Machado C.d.C., Bugarin A.d.S., Ma-chado I.F., Costa I. (2020). Exfoliation and intergranular corrosion resistance of the 2198 Al-Cu-Li alloy with different thermomechanical treatments. Mater. Corros..

[28] Wang J., Zhang B., Wu B., Ma X. (2016). Size-dependent role of S phase in pitting initiation of 2024Al alloy. Corros. Sci..

[29] Ma S., Zhao Y., Zou J., Yan K., Liu C. (2017). The effect of laser surface melting on microstructure and corrosion behavior of friction stir welded aluminum alloy 2219. Opt. Laser Technol..

[30] Liu Z., Chong P.H., Skeldon P., Hilton P.A., Spencer J.T., Quayle B. (2006). Fundamental understanding of the corrosion performance of laser-melted metallic alloys. Surf. Coat. Technol..

[31] Surekha K., Murty B., Rao K.P. (2009). Effect of processing parameters on the corrosion behaviour of friction stir processed AA 2219 aluminum alloy. Solid State Sci..

[32] Lu Y., Wang J., Li X., Li W., Li R., Zhou D. (2018). Effects of pre-deformation on the microstructures and corrosion behavior of 2219 aluminum alloys. Mater. Sci. Eng. A.

[33] Huang J.L., Li J.F., Liu D.Y., Zhang R.F., Chen Y.L., Zhang X.H., Ma P.C., Gupta R.K., Birbilis N. (2018). Correlation of inter-granular corrosion behaviour with microstructure in Al-Cu-Li alloy. Corros. Sci..

[34] Grilli R., Baker M.A., Castle J.E., Dunn B., Watts J.F. (2010). Localized corrosion of a 2219 aluminium alloy exposed to a 3.5% NaCl solution. Corros. Sci..

[35] Chen Y., Zhou J., Liu C., Wang F. (2018). Effect of pre-deformation on the pre-corrosion multiaxial fatigue behaviors of 2024-T4 aluminum alloy. Int. J. Fatigue.

[36] Hikku G., Jeyasubramanian K., Venugopal A., Ghosh R. (2017). Corrosion resistance behaviour of graphene/polyvinyl alcohol nanocomposite coating for aluminium-2219 alloy. J. Alloy. Compd..

[37] Kairy S.K., Rouxel B., Dumbre J., Lamb J., Langan T.J., Dorin T., Birbilis N. (2019). Simultaneous improvement in corrosion re-sistance and hardness of a model 2xxx series Al-Cu alloy with the microstructural variation caused by Sc and Zr additions. Corros. Sci..

[38] Zhu Z., Deng C., Wang Y., Yang Z., Ding J., Wang D. (2015). Effect of post weld heat treatment on the microstructure and corrosion behavior of AA2219 aluminum alloy joints welded by variable polarity tungsten inert gas welding. Mater. Des..

[39] Emarati S.M., Mozammel M. (2020). Theoretical, fundamental and experimental study of liquid-repellency and corrosion resistance of fabricated superamphiphobic surface on Al alloy 2024. Chem. Eng. J..

[40] Simsek I., Nalcacioglu C., Ozyurek D. (2019). The effects of aging temperature on the corrosion and electrical conductivity in the AA7075 alloy produced by powder metallurgy method. Acta Phys. Pol. A.

[41] Shi T., Li X., Zhang Q., Li B. (2018). One-step potentiostatic deposition of micro-particles on Al alloy as superhydrophobic surface for enhanced corrosion resistance by reducing interfacial interactions. Coatings.

[42] Yang Q., Zhou Y., Li Z., Mao D. (2019). Effect of hot deformation process parameters on microstructure and corrosion behavior of 35CrMoV steel. Materials.

[43] Soleimani M., Mirzadeh H., Dehghanian C. (2020). Processing route effects on the mechanical and corrosion properties of dual phase steel. Met. Mater. Int..

[44] Sun Y., Pan Q., Sun Y., Wang W., Huang Z., Wang X., Hu Q. (2019). Localized corrosion behavior associated with Al7Cu2Fe intermetallic in Al-Zn-Mg-Cu-Zr alloy. J. Alloy. Compd..

[45] Lin Y.-C., Zhang J.-L., Chen M.-S., Zhou Y., Ma X. (2016). Electrochemical corrosion behaviors of a stress-aged Al-Zn-Mg-Cu alloy. J. Mater. Res..

[46] Xu L., Zhang J., Han Y., Zhao L., Jing H. (2019). Insights into the intergranular corrosion of overlay welded joints of X65-Inconel 625 clad pipe and its relationship to damage penetration. Corros. Sci..

[47] Fadaee H., Javidi M.M. (2014). Investigation on the corrosion behaviour and microstructure of 2024-T3 Al alloy treated via plasma electrolytic oxidation. J. Alloy. Compd..

[48] Shaik M.A., Syed K.H., Golla B.R. (2019). Electrochemical behavior of mechanically alloyed hard Cu-Al alloys in marine environment. Corros. Sci..

[49] Soleimani M., Mirzadeh H., Dehghanian C. (2021). Effects of spheroidization heat treatment and intercritical annealing on mechanical properties and corrosion resistance of medium carbon dual phase steel. Mater. Chem. Phys..

[50] Shi W., Zhou H., Zhang X. (2019). High-strength and anti-corrosion of Al-Cu-Mg alloy by controlled ageing process. Philos. Mag. Lett..

[51] Lin Y.-C., Jiang Y.-Q., Zhang J.-L., Chen X.-M. (2018). Influence of stress-aging processing on precipitates and mechanical properties of a 7075 aluminum alloy. Adv. Eng. Mater..

[52] Deng Y.L., Yin Z.M., Zhao K., Duan J.Q., Hu J., He Z.B. (2012). Effects of Sc and Zr microalloying additions and aging time at 120 C on the corrosion behaviour of an Al-Zn-Mg alloy. Corros. Sci..

[53] Wen D., Long P., Li J., Huang L., Zheng Z. (2020). Effects of linear heat input on microstructure and corrosion behavior of an austenitic stainless steel processed by wire arc additive manufacturing. Vacuum.

[54] Lin Y.-C., Liu G., Chen M.-S., Huang Y.-C., Chen Z.-G., Ma X., Jiang Y.-Q., Li J. (2016). Corrosion resistance of a two-stage stress-aged Al-Cu-Mg alloy: Effects of stress-aging temperature. J. Alloy. Compd..

[55] Khan M.A., Wang Y., Anjum M.J., Yasin G., Malik A., Nazeer F., Khan S., Ahmad T., Zhang H. (2020). Effect of heat treatment on the precipitate behaviour, corrosion resistance and high temperature tensile properties of 7055 aluminum alloy synthesis by novel spray deposited followed by hot extrusion. Vacuum.

[56] Zhan X., Tang J., Li H., Liang X., Lu Y., Che Y., Tu W., Zhang Y. (2020). Effects of non-isothermal aging on mechanical properties, corrosion behavior and microstructures of Al-Cu-Mg-Si alloy. J. Alloy. Compd..

[57] Azarniya A., Taheri A.K., Taheri K.K. (2019). Recent advances in ageing of 7xxx series aluminum alloys: A physical metallurgy perspective. J. Alloy. Compd..

[58] Shao L., Li H., Jiang B., Liu C., Gu X., Chen D. (2018). A comparative study of corrosion behavior of hard anodized and micro-arc oxidation coatings on 7050 aluminum alloy. Metals.

[59] Saillard R., Viguier B., Odemer G., Pugliara A., Fori B., Blanc C. (2018). Influence of the microstructure on the corrosion behaviour of 2024 aluminium alloy coated with a trivalent chromium conversion layer. Corros. Sci..

[60] Ferri M., Trueba M., Trasatti S., Cabrini M., Conte A.L. (2017). Electrochemical investigation of corrosion and repassivation of structural aluminum alloys under permanent load in bending. Corros. Rev..

[61] Trueba M., Trasatti S. (2018). The repassivation response from single cycle anodic polarization: The case study of a sensitized Al-Mg alloy. Electrochim. Acta.

[62] Sun S., Fang Y., Zhang L., Li C., Hu S. (2020). Effects of aging treatment and peripheral coarse grain on the exfoliation corrosion behaviour of 2024 aluminium alloy using SR-CT. J. Mater. Res. Technol..

[63] Su R., Wang K., Yang Y., Qu Y., Li R. (2020). Effect of Mg content on the microstructure and corrosion properties of Al-Cu-Mn alloy. J. Mater. Eng. Perform..

[64] Kaithwas C., Bhuyan P., Pradhan S., Mandal S. (2018). Microstructure evolution during low-strain thermo-mechanical processing and its repercussion on intergranular corrosion in alloy 600H. Mater. Charact..

[65] Pradhan S., Bhuyan P., Mandal S. (2018). Individual and synergistic influences of microstructural features on intergranular corrosion behavior in extra-low carbon type 304L austenitic stainless steel. Corros. Sci..

[66] Deepak K., Mandal S., Athreya C.N., Kim D.I., Boer B.D., Sarma V.S. (2016). Implication of grain boundary engineering on high temperature hot corrosion of alloy 617. Corros. Sci..

[67] Wang Z., Chen P., Li H., Fang B., Song R., Zheng Z. (2017). The intergranular corrosion susceptibility of 2024 Al alloy during re-ageing after solution treating and cold-rolling. Corros. Sci..

[68] Li B., Pan Q.-L., Chen C.-P., Yin Z. (2016). Effect of aging time on precipitation behavior, mechanical and corrosion properties of a novel Al-Zn-Mg-Sc-Zr alloy. Trans. Nonferr. Met. Soc..

[69] Du J., Ding D., Zhang W., Xu Z., Gao Y., Chen G., You X., Chen R., Huang Y., Tang J. (2018). Effect of Ce addition on the microstructure and properties of Al-Cu-Mn-Mg-Fe lithium battery shell alloy. Mater. Charact..

[70] Huang L., Chen K., Li S. (2012). Influence of grain-boundary pre-precipitation and corrosion characteristics of inter-granular phases on corrosion behaviors of an Al-Zn-Mg-Cu alloy. Mater. Sci. Eng. B.

